# International Society of Gynecological Pathologists (ISGyP) Endometrial Cancer Project: Guidelines From the Special Techniques and Ancillary Studies Group

**DOI:** 10.1097/PGP.0000000000000496

**Published:** 2018-12-14

**Authors:** Kathleen R. Cho, Kumarasen Cooper, Sabrina Croce, Bojana Djordevic, Simon Herrington, Brooke Howitt, Pei Hui, Philip Ip, Martin Koebel, Sigurd Lax, Bradley J. Quade, Patricia Shaw, August Vidal, Anna Yemelyanova, Blaise Clarke, Lora Hedrick Ellenson, Teri A. Longacre, Ie-Ming Shih, W. Glenn McCluggage, Anais Malpica, Esther Oliva, Vinita Parkash, Xavier Matias-Guiu

**Affiliations:** MD Anderson Cancer Center, Houston, Texas (R.B., A.Y., A.M.); University of Michigan Medical School, Ann Arbor, Michigan (K.R.C.); Hospital of the University of Pennsylvania, Philadelphia, Pennsylvania (K.C.); Bergonie Institute, Bordeaux, France (S.C.); The Ottawa Hospital, University of Ottawa, Ottawa (B.D.); Toronto General Hospital, Toronto (P.S., B.C.), Ontario; Department of Pathology, University of Calgary, Calgary, Alberta (M.K.), Canada; Edinburgh Cancer Research Centre, Edinburgh (S.H.); Belfast Health and Social Care Trust, Belfast (W.G.M.), UK; Brigham and Women’s Hospital (B.H., B.J.Q.); Massachusetts General Hospital, Harvard University (E.O.), Boston, Massachusetts; Yale New Haven Hospital, New Haven, Connecticut (P.H., V.P.); Department of Pathology, University of Hong Kong, Hong Kong, China (P.I.); Department of Pathology, Medical University of Graz, Graz, Austria (S.L.); Bellvitge University Hospital, IDIBELL, Barcelona (A.V., X.M.G.); Hospital Universitari Arnau de Vilanova, University of Lleida, IRBLLEIDA, Lleida (X.M.G.), CIBERONC, Spain; Weill Cornell Medical College, New York, New York (L.H.E.); Stanford University Medical Center, Stanford, California (T.A.L.); Johns Hopkins University School of Medicine, Baltimore, Maryland (I.M.S.)

**Keywords:** Endometrial carcinoma, Guidelines, ISGyP, Molecular pathology

## Abstract

The aim of this article is to propose guidelines and recommendations in problematic areas in pathologic reporting of endometrial carcinoma (EC) regarding special techniques and ancillary studies. An organizing committee designed a comprehensive survey with different questions related to pathologic features, diagnosis, and prognosis of EC that was sent to all members of the International Society of Gynecological Pathologists. The special techniques/ancillary studies group received 4 different questions to be addressed. Five members of the group reviewed the literature and came up with recommendations and an accompanying text which were discussed and agreed upon by all members of the group. Twelve different recommendations are made. They address the value of immunohistochemistry, ploidy, and molecular analysis for assessing prognosis in EC, the value of steroid hormone receptor analysis to predict response to hormone therapy, and parameters regarding applying immunohistochemistry and molecular tests for assessing mismatch deficiency in EC.

In the western world, endometrial carcinoma (EC) is the fourth most common cancer among women, with an estimated incidence of 10 to 20 per 100,000 women. Although the prognosis is favorable for patients with low-grade tumors and early-stage disease, the outcomes for patients with high-grade and/or advanced stage tumors remains relatively poor. Following surgery (which is the usual treatment), patients with tumors with a high risk of recurrence often receive adjuvant radiotherapy, chemotherapy, and/or hormone therapy. However, traditional chemotherapeutic regimes are less effective in comparison with cancers of other organs, which emphasize the importance of identifying new molecular targets. Several biomarkers have been proposed to better assess the prognosis and prediction of response to therapy and to identify patients carrying germline mutations associated with an increased risk of EC 1. However, there is no consensus regarding the application of these biomarkers in clinical practice.

In this article, we provide guidelines, which have been produced after a thorough literature search, and discussed among a group of members of the International Society of Gynecological Pathologists (ISGyP), selected because of their expertise in the immunohistochemistry (IHC) and molecular pathology of EC.

## METHODS

An organizing committee composed of 5 of the authors (W.G.M., V.P., A.M., E.O., and X.M.G.) was chosen by the Executive Committee of ISGyP. The committee designed a survey (see paper on survey) with many different questions, related to the pathologic features, diagnosis, and prognosis of EC, which was sent to all members of ISGyP. After analysis of the responses, the organizing committee selected several problematic areas that would be addressed, to achieve consensus and provide recommendations. The organizing committee created 3 different groups: (1) diagnosis, (2) processing/sampling/staging/prognosis, and (3) special techniques/ancillary studies. Several questions were sent to each group.

The special techniques/ancillary studies group addressed 4 different questions. Five members of the group (T.A.L., B.C., I.M.S., L.H.E., and X.M.G.) reviewed the literature and came up with recommendations and accompanying text which were discussed and agreed by all members of the group. Levels of evidence and strength of the recommendations were established according to criteria established in Table 1.

**TABLE 1:**
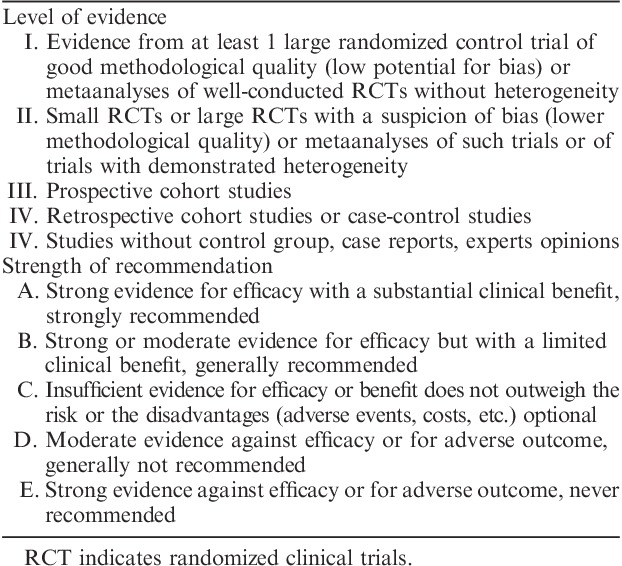
Level of evidence and strength of recommendation adapted from ESMO 2014 endometrial cancer consensus conference

### Question 1. Should IHC and Ploidy Analysis be Performed for Assessing Prognosis in EC?

A preliminary comment is necessary to emphasize that IHC is important in histologic typing, and this is relevant for prognosis. This is particularly important for high-grade EC, including high-grade endometrioid carcinoma (EEC), serous carcinoma (SC), and clear cell carcinoma, since there is poor interobserver agreement in diagnosing this subset of tumors 2. These issues will be addressed by the diagnosis group.

Other than markers which are useful in diagnosis, there are few specific studies that provide definitive evidence for the routine use of IHC or ploidy analysis in determining the prognosis of EC.

There has been considerable literature on the association between prognosis and the IHC analysis of estrogen receptor (ER), progesterone receptor (PR), Ki67, and p53 in EC 3. It is well established that there is an association between the expression of these markers and prognosis and there is a significant literature regarding this 4–7, but there are a lack of large prospective studies to determine their prognostic utility.

Hormone receptor status has been suggested to be a relevant prognostic marker, and the presence of steroid receptors correlates with low tumor grade, as well as favorable outcome in some studies. However, there is no absolute evidence that steroid hormone receptor analysis should be incorporated into clinical practice.

Similar comments pertain to ploidy analysis. There is some literature on the association of ploidy with prognosis, with promising results 8,9, but there is a lack of definitive studies to determine its true prognostic impact 10.

There are a number of additional potential biomarkers reported for EC, but none of them are validated for use as robust prognostic indicators. New prognostic markers such as L1CAM 11,12 or Annexin A-2 13 are promising, but require prospective validation studies before bringing them into clinical practice. Additional potentially useful markers are intratumoral lymphocytes (CD8+), tumor-associated macrophages, loss of ASRGL1, or hyaluronidase 1 14,15. There is particular interest regarding L1CAM and several studies have validated this marker in multicentric analysis 16,17.

One of the important issues with implementing these markers in the routine clinical setting is the lack of uniformity regarding the methodology used, both technical and interpretive. Clearly, large prospective, well defined, uniform studies are needed to determine the possible role of IHC for specific biomarkers and ploidy analysis in the clinical setting.

### Question 2. Should ER IHC be Used to Predict Response to Hormone Therapy? Are There Any Other Predictive Markers of Response to Hormone Therapy?

Hormone therapy is sometimes administered to patients with advanced or recurrent EC, particularly low-grade EEC 18. A wide range of hormonal agents have been used, including medroxyprogesterone acetate and synthetic progestational agents, LHRH antagonists, tamoxifen, and new generations of selective estrogen receptor modulators 19.

Although receptor IHC estimation has not been universally accepted as the standard for prediction of hormone response, several international guidelines recommend determination of hormone receptor status before hormone therapy is initiated 18.

A recent systematic review of 5 randomized trials and 29 phase II studies, comprising a total of 2471 patients, concluded that hormone receptor assessments should be carried out in all patients entered into clinical trials, and may aid clinical management in selected patients 20. The report notes that receptor-negative status is not an absolute contraindication to hormone treatment. Response rates to various hormonal treatments for EC patients are higher for patients with low-grade EEC, and those with PR expression. However, the methodology for assessing and scoring hormone receptor expression in EC was variable in the reported series.

Furthermore, changes in ER, and particularly PR, expression occur during tumor progression, and expression is generally higher in primary in comparison with metastatic tumors. This suggests that assessment of ER status in the primary tumor may not reflect the status in the recurrent or metastatic tumor and that biopsies of recurrent or metastatic tumors with hormone receptor analysis on these may be helpful 21.

In summary, at the present time there is not enough data to support that the ER or PR status of tumors, as determined by IHC, is a reliable marker for predicting response to hormone therapy. There are studies that have concluded that positive staining does correlate with response, but other studies have indicated that there is no correlation. Large prospective studies using defined, uniform approaches need to be performed to determine whether ER and/or PR IHC is a robust marker to predict hormone therapy response.

### Question 3. Should Molecular Analysis be Performed to Diagnose and/or Classify Appropriately EC? When Should This be Used and Which Analysis is Recommended?

Tests based on sets of genes that are differentially expressed in ECs compared with normal endometrium cells have been proposed as adjuncts to diagnosis on endometrial biopsies. They have been shown to be useful in women with suspicion of cancer and noninformative endometrial biopsies with insufficient material to allow a specific diagnosis.

Although promising, and assessed in prospective studies 22, their incorporation into clinical practice requires further validation.

There are different pathologic variants of EC. Single-gene approaches have shown that the molecular alterations involved in the development of EEC are different from those of SC, clear cell carcinoma, and carcinosarcoma. Many EECs exhibit microsatellite instability (MSI), as well as mutations in *PTEN*, *KRAS*, and *CTNNB1* whereas SC exhibits alterations of *TP53*, widespread loss of heterozygosity, as reflected by chromosomal instability, as well as other molecular alterations 1.

The Cancer Genome Atlas (TCGA) Research Network performed an integrated genomic characterization of EC 23. The genes most frequently mutated in EEC were *PTEN* (77%), *PIK3CA* (53%), *PIK3R1* (37%), *CTNNB1* (36%), *ARID1A* (35%), *KRAS* (24%), *CTCF* (20%), *RPL22* (12%), *TP53* (11%), *FGFR2* (11%), and *ARID5B* (11%). The genes most frequently mutated in SC were *TP53* (90.7%), *PIK3CA* (41.9%), *FBXW7* (30.2%), *PPP2R1A* (36.6%), *CHD4* (16.3%), *CSMD3* (11.6%), and *COLA 11* (11.6%) 1. Additional studies using exome-sequencing analysis have also shown mutations in *TAF-1* (30%), in SC 24.

In addition to the TCGA approach, several groups have assessed the usefulness of next-generation sequencing in differential diagnosis between different types of tumors, particularly EEC and SC 25. This may be particularly interesting in tumors with noninformative immunohistochemical results and ambiguous microscopic appearances. Further studies are required to investigate whether next-generation sequencing is more accurate, informative, clinically relevant, and cost-effective in comparison with IHC.

The TCGA exome sequence analysis revealed 4 groups of tumors with significant differences in prognosis. Group 1 comprised EEC with mutations in *POLE* (ultramutated), associated with good prognosis. Group 2 comprised EEC with MSI (hypermutated) and group 3 tumors comprised EEC with low copy number alterations; both groups 2 and 3 tumors showed similar and intermediate progression-free survival rates. Group 4 (serous-like) tumors show *TP53* mutations, and a worse prognosis. They are composed mostly of SCs, but also include some EEC (many grade 3 but also some grades 1 and 2).

Following the TCGA study, sequencing of *POLE* has been proposed to identify the group of ultramutated tumors, which have been associated with an excellent prognosis in several case series 26,27. *POLE* sequencing seems to be particularly relevant in the subgroup of patients with high-grade EEC, particularly in those with some features mimicking SC 28, although SC may also show *POLE* mutations 29. Incorporation of *POLE* sequencing in the assessment of endometrial tumors may have an impact in tailoring treatment and possibly reducing the extent of surgery, in a subset of patients with high-grade ECs 30,31. Prospective studies are recommended to assess the benefits and cost-effectiveness of *POLE* sequencing. Development of molecular tests for *POLE* analysis is encouraged, particularly in pathology departments with experience in molecular pathology. Identification of possible IHC surrogates of *POLE* mutation in EC may also be of great interest.

Combining *POLE* mutational analysis with IHC analysis of p53 and mismatch repair (MMR) proteins (PMS-2 and MSH-6) has been proposed to classify ECs into the 4 TCGA groups. This is likely to be particularly useful for high-grade EEC and SC, as a surrogate assay that can replicate TCGA classification 30–32 (Table 2).

**TABLE 2:**
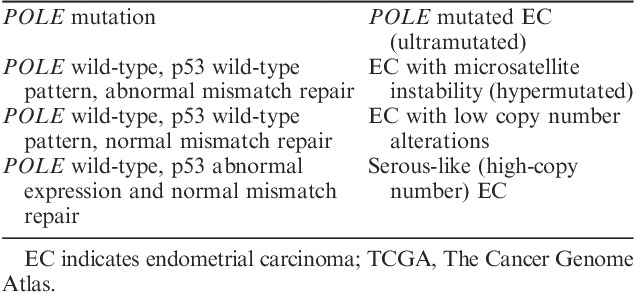
Surrogate approach for applying TCGA scheme into pathology practice

This topic will be also analyzed in another manuscript. However, it is worth mentioning that there are some aspects that should be taken into account. For example, not all *POLE-*mutated EC are hypermutated. Moreover, by performing MMR proteins IHC first, we will be missing some number of *POLE*-mutated cases. Interpretation of p53 immunostaining will be also discussed in a separate manuscript.

It is important to note that some tumors have intermediate features (double mutant), and the prognostic relevance of these unusual patterns will require further analysis. Application of TCGA surrogate classification to high-grade EC may also potentially help in deciding whether immunotherapy is useful 33. Emerging data have suggested that EECs exhibiting POLE mutations and MSI are hypersensitive to the immune checkpoint inhibitor, anti-PD-1, monotherapy because these tumors are characterized by a high mutation load which produces more neo-antigens. They also have a higher number of tumor infiltrating lymphocytes 34–36. There is a report of 2 cases of EC (1 *POLE* mutated, 1 *MSH-6* mutated) both of whom were refractory to chemotherapy and radiation therapy, who both responded (sustained partial response by RECIST criteria) to nivolumab 37.

Addition of *CTNNB1* (the gene coding for beta-catenin) mutation analysis has also been proposed in EEC with low copy number alteration, as a prognostic biomarker in this subset of tumors 38.

Recent preclinical studies also demonstrate that tumors with *ARID1A* mutations are more sensitive to PARP1 inhibitors and EZH2 inhibitors 39,40. If the results of future clinical trials show clinical benefit, analyzing ARID1A expression status (loss of expression is a surrogate for *ARID1A* inactivating mutation) in EEC could be useful to stratify patients for ARID1A-based therapy.

### Question 4. Should IHC and/or MSI (Including Methylation Analysis) be Performed Routinely in Apparently Sporadic ECs? All Cases? When?

Approximately 3% to 5% of ECs can be attributed to Lynch syndrome (LS) which is caused by germline mutations in DNA MMR genes (*MLH1*, *MSH-2*, *MSH-6*, *PMS-2*). Patients with LS have a 40% to 60% lifetime risk for endometrial and colon cancer 41–43. The identification of LS in women with EC can lead to the prevention of a second cancer in the patient and incident cancers in family members through risk-reducing strategies and heightened surveillance 44,45. Utilization of clinical criteria to identify patients with LS has less than optimal sensitivity and efficiency.

A Society of Gynecologic Oncologists Clinical Practice Statement in 2014 recommended all women diagnosed with EC undergo systematic clinical screening (review of personal and family history) and/or molecular or IHC screening for LS 46. In this statement IHC screening of EC for LS is stated to be the preferred screening strategy when resources are available for the following reasons.

Systematic clinical screening, including a focused personal and family history, will miss a significant fraction of women with LS who do not have a suggestive family history 47–51. Some studies indicate that up to 75% of LS patients are missed using the revised Bethesda guidelines. It has been proposed that the more sensitive strategy involves universal tumor testing for either all endometrial cancers or cancers diagnosed at age <60 (or 70), regardless of personal or family cancer history. Although EC can be screened for LS using IHC for the 4 MMR proteins (MLH1, MSH-2, MSH-6, PMS-2) and/or MSI analysis, with reflex *MLH1* hypermethylation testing, IHC is the most cost-effective method and is widely available in almost all pathology laboratories. IHC testing of tumor tissue for lack of expression of MMR proteins has an overall reported sensitivity and specificity for LS of 83% and 89%, respectively. Additional advantages of IHC testing include (1) absence of expression of a specific mismatch protein can direct germline testing to that specific gene and (2) the relative increased prevalence of *MSH-6* mutations in EC which may not exhibit MSI by molecular analysis. The specificity of both MSI and IHC testing in the detection of LS decreases with increasing age due to increased prevalence of somatic *MLH1* hypermethylation 52–55.

As the majority of cases with MLH1 IHC loss are due to *MLH1* hypermethylation, a sporadic cause of MLH1 loss, *MLH1* hypermethylation analysis should be undertaken on tumors that show IHC loss of MLH1 to help triage appropriate cases for germline testing. It is well documented that *MLH1* promoter hypermethylation analysis is an accurate, cost-effective, and superior prescreening method compared with *BRAF* mutation analysis in the diagnostic algorithm of LS for EC. *MLH1* gene promoter hypermethylation can be undertaken at low costs in a routine molecular diagnostic setting, for example, by methylation-specific polymerase chain reaction or by methylation-specific multiplex ligation-dependent probe amplification. Given the inclusion of patient-derived normal DNA in the *MLH1* promoter hypermethylation, the rare occurrence of germline *MLH1* hypermethylation can also be detected. The most cost-effective approach involves screening with MSH-6 and PMS-2 IHC alone, with subsequent undertaking of MSH-2 and/or MLH1 IHC and reflex *MLH1* hypermethylation when indicated.

The rationale behind universal testing is to reduce the morbidity and mortality of index patients as well as relatives of patients with LS. Universal testing for LS in colorectal cancer (CRC) has been endorsed by the National Comprehensive Cancer Network. Evaluation of a universal strategy by Ladabaum et al. 56 revealed that a systematic application of testing among patients with newly diagnosed CRC at ≤70 yr of age could provide substantial clinical benefits at acceptable costs. Other studies have also reported the cost-effectiveness of universal CRC testing 56. Ladabaum et al. 56 concluded that IHC testing of CRC for MMR proteins followed by reflex testing of the tumors when MLH1 protein expression is absent emerged as the most cost-effective approach. These data are not as robust for EC, but they appear to parallel that for CRC 56.

Approximately 50% of patients with EC with MMR deficiency (not due to *MLH1* hypermethylation) do not harbor an apparent germline mutation. The cause of such discordance can be attributed to unidentified germline mutations, a false positive MMR deficiency test, or biallelic somatic inactivation of the MMR gene(s). Recent evidence based on next-generation sequencing suggests that almost 70% of these tumors harbor biallelic acquired somatic (tumor) mutations and/or loss of heterozygosity in MMR genes 57,58. Given the screening implications associated with a LS-suspected tumor, somatic mutational analysis and loss of heterozygosity should be considered in the diagnostic algorithm.

Although universal screening testing of EC for LS is suggested, development and implementation of screening programs are complicated. These programs require cooperation and effective communication across multiple disciplines, ensuring that patients at risk for LS are identified, notified of abnormal results, and referred for genetic counseling and genetic testing. Moreover, the accuracy of IHC is operator dependent and varies according to the experience and skill of the laboratory performing the testing. Consequently, it may be that testing is optimally performed in laboratories with high volume and high-quality control measures.

Panel testing for germline mutations in >20 cancer-causing genes (which include the MMR and *EPCAM* genes) is now available commercially as a single test. Inevitably, advances in technology will decrease the cost of such analysis. In the future, germline testing, rather than tumor evaluation, may be the most cost-effective universal testing approach.

As per the recommendations of the Society of Gynecologic Oncologists, testing all patients with EC for LS is recommended. If utilizing this strategy, most experts would recommend routine tumor-based testing on ECs with IHC followed by methylation testing, if there is loss of expression of MLH1. Alternatively, the EC can be initially tested for MSI, but any abnormality would require the additional step of IHC and most experts agree that molecular testing by polymerase chain reaction misses many MSH-6 deficient tumors. Universal tumor testing is likely to become the future international standard of care and is already conducted in some hospitals in United States and elsewhere. However, this standard requires development of sufficient local and community infrastructure to appropriately handle genetic results before implementation, as discussed previously. Consequently, testing could be considered for all patients with EC 70 yr of age or younger (in corollary to the CRC recommendations) when appropriate infrastructure for testing exists. If tumor testing is performed for those aged 70 yr or younger only, a thorough family history is essential for those EC patients older than 70 yr; IHC testing should be performed for any individual whose personal and/or family history fulfill the Amsterdam or Bethesda guidelines or who have a ≥5% risk prediction based on the prediction models.

Independent of LS screening, the identification of the MSI (hypermutated)/MMR deficient phenotype in colorectal (and other) carcinomas has been recently exploited for targeted immune therapy with anti-PD-1, as has been mentioned previously. Potential applications for such targeted therapy in EC have not been as well investigated, but remain a viable possibility 59,60.

## SUMMARY OF RECOMMENDATIONS

### Recommendation 1

The prognostic value of IHC and DNA ploidy in EC is not clear. It is recommended to perform large prospective, multicenter studies with good methodological quality, to definitely prove the potential prognostic utility of these tests.

**Level of evidence: IV**

**Strength of recommendation:** C

### Recommendation 2

The predictive value of ER and PR IHC in response to hormone therapy in EC (particularly low-grade EEC) is not clear. It is recommended to perform large prospective, multicenter studies with good methodological quality, to definitely prove its potential predictive value. This should be done, by taking into account the following variables:

- Different drugs used.
- Differences between low-grade and high-grade tumors.
- Different methodological and scoring approaches.
- Best tissue type (primary vs. metastatic tumor).

**Level of evidence: II**

**Strength of recommendation:** C

### Recommendation 3

Validation of molecular tests for diagnosis of EC is encouraged as an adjunct to diagnosis in endometrial biopsies. This is particularly potentially interesting in women with a suspicion of EC and noninformative endometrial biopsies.

**Level of evidence: IV**

**Strength of recommendation:** B

### Recommendation 4

Investigational molecular studies are encouraged to assess the value of next-generation sequencing in differential diagnosis between different types of EC, by considering the potential benefits and cost-effectiveness in comparison with IHC and morphologic analyses.

**Level of evidence: IV**

**Strength of recommendation:** C

### Recommendation 5

Mutational analysis of *POLE* is considered optional for tailoring treatment in patients with high-grade EC.

**Level of evidence: IV**

**Strength of recommendation:** A

### Recommendation 6

Combining *POLE* mutational analysis with IHC analysis of p53 and MMR proteins (PMS-2 and MSH-6) is considered optional as a surrogate to classify tumors into the 4 TCGA groups, particularly for high-grade EEC and SC.

**Level of evidence: IV**

**Strength of recommendation:** A

### Recommendation 7

Development of simple molecular tests for *POLE* analysis or IHC surrogates of *POLE* mutation, is encouraged to allow POLE assessments in pathology departments with limited molecular pathology facilities.

**Level of evidence: IV**

**Strength of recommendation:** C

### Recommendation 8

All women who are diagnosed with EC should undergo systematic clinical screening for LS (review of personal and family history) and/or IHC/molecular screening.

**Level of evidence:** III

**Strength of recommendation:** B

### Recommendation 9

IHC screening of EC for LS is the preferred strategy when resources are available. The most cost-effective approach involves screening by MSH-6 and PMS-2 IHC alone, with subsequent MSH-2 or MLH1 IHC when indicated.

**Level of evidence:** III

**Strength of recommendation:** B

### Recommendation 10

*MLH1* hypermethylation analysis should be completed on tumors that show loss of MLH1 on IHC to help triage appropriate cases for germline testing.

**Level of evidence:** I

**Strength of recommendation:** A

### Recommendation 11

Because of the occurrence of potential somatic mutations in MMR genes, somatic mutation analysis should be considered if germline testing in appropriately triaged patients is negative.

**Level of evidence:** IV

**Strength of recommendation:** B

### Recommendation 12

As women with EC with MMR deficiency or MSI (hypermutated) may benefit from immunotherapy, consideration should be given for MMR deficiency testing for women with EC who are candidates for chemotherapy.

**Level of evidence:** IV

**Strength of recommendation:** B
